# CT coronary angiography in the lipid clinic: a pilot study and lipidologist survey

**DOI:** 10.1007/s10554-025-03526-3

**Published:** 2025-10-09

**Authors:** John Graby, James Sellek, Ali Khavandi, Dylan Thompson, Will W. Loughborough, Benjamin J. Hudson, Tony Avades, Wycliffe Mbagaya, Ahai Luva, Nigel Capps, Cheerag Shirodaria, Graham Bayly, Charalambos Antoniades, Paul F. Downie, Jonathan C. L. Rodrigues

**Affiliations:** 1 Department of Cardiology, Royal United Hospitals Bath NHS Foundation Trust, Combe Park, Bath, Avon BA1 3NG UK; 2 Department for Health, University of Bath, Claverton Down, Bath, BA2 7AY UK; 3 Department of Radiology, Royal United Hospitals Bath NHS Foundation Trust, Combe Park, Bath, Avon BA1 3NG UK; 4 Department of Chemical Pathology, University Hospitals Plymouth NHS Trust, Plymouth, PL6 8DH UK; 5 Department of Clinical Biochemistry, University Hospitals Bristol and Weston NHS Trust, Bristol, BS2 8HW UK; 6 Department of Clinical Biochemistry, The Newcastle upon Tyne Hospitals NHS Foundation Trust, Freeman Rd, High Heaton, Newcastle upon Tyne, NE7 7DN UK; 7 Department of Chemical Pathology, The Shrewsbury and Telford Hospital NHS Trust, Princess Royal Hospital, Telford, TF1 6TF UK; 8 Oxford University Hospitals NHS Foundation Trust, Headley Way, Headington, Oxford, OX3 9DU UK; 9 Acute Multidisciplinary Imaging & Interventional Centre, University of Oxford, John Radcliffe Hospital, Oxford, OX3 9DU UK

**Keywords:** Hyperlipidaemias, Coronary artery disease, Computed Tomography Angiography, Cardiac Imaging Techniques, Heart disease risk factors

## Abstract

Guidelines recommend considering coronary calcium score (CCS) in asymptomatic patients to aid risk stratification. However, calcification occurs late in atherosclerosis. Coronary CT angiography (CCTA) can detect non-calcific plaque and inflammation before calcification develops, but impact on clinical management is not well documented. We compare coronary artery disease (CAD) detection and grading between CCS and CCTA, impact on management, and explore CCTA-derived inflammation biomarker (pericoronary fat attenuation index [FAI]) in the lipid clinic. Exploratory analysis of a prospectively maintained database of lipid clinic patients with CCS and CCTA (2018–2020). CCS grade was compared with CCTA stenosis, presence of high-risk plaque (HRP) and FAI-score analysis. UK Consultant Lipidologists completed an anonymised survey, documenting lipid target and management after sequential unblinding of CCS and CCTA data. In 45 asymptomatic patients (49% female, mean age 55 ± 9), CCTA re-classified CAD presence in 22% (*p* = 0.002) and severity in 62% (*p* = 0.005) vs. CCS. HRP was observed in 20% (9/45), including 56% with CCS ≤ 100. Median LDL target with clinical vignette was 101 mg/dL (IQR 77–120), reducing to 89 mg/dL (77–120) after CCS, and 77 mg/dL (70–116) after CCTA unblinding. CCS altered LDL target in 12%, and CCTA a further 19% (χ2 57.0, *p* < 0.005). High FAI-score was demonstrated in 20%, including 22% of those with CCS ≤ 100 and 75% of those with ≤ mild CAD on CCTA. CCTA increased CAD prevalence and re-classified severity versus CCS, altering hypothetical management. High FAI-scores were observed across CCS and CCTA severity grades, including patients with no overt CAD.

**Supplementary Information:**

The online version contains supplementary material available at 10.1007/s10554-025-03526-3.

## Introduction

Dyslipidaemia promotes atherosclerosis and is an important risk factor for major adverse cardiovascular events (MACE) [1]. Genetic forms of dyslipidaemia, for example familial hypercholesterolaemia (FH), are associated with a significantly heightened risk of coronary artery disease (CAD) and premature MACE [2]. However, this risk is both heterogeneous within FH and other lipid diagnoses managed in the lipid clinic, and modifiable where appropriate treatment is instituted early [3, 4]. Indeed, new data suggest that early and intensive lipid-lowering substantially reduces long-term risk, reinforcing the “earlier and lower is better” paradigm [5]. As such, international guidance recommends cardiovascular risk stratification in all individuals, establishing lipid targets and treatment recommendations to achieve these [6–10].

In the primary prevention setting, the presence of proven atherosclerotic cardiovascular disease (ASCVD) escalates risk with associated lowering of low-density lipoprotein (LDL) targets and implications for recommended lipid-modifying therapies. This holds true even in the higher cardiovascular risk cohort seen in the lipid clinic.

Non-invasive cardiovascular imaging can detect and quantify subclinical atherosclerosis regardless of symptoms. The impact this has on clinical management in the lipid clinic is not fully understood. While coronary calcium scoring (CCS) remains a first-line tool in asymptomatic patients and provides a surrogate of atherosclerotic burden linked to MACE risk [7, 11–13], its limitations are well documented. Specifically, calcification reflects late-stage atherosclerosis [14], whilst statin therapy may paradoxically increase CCS, complicating the interpretation of serial scans [15]. Moreover, CCS tends to be low among patients aged under 45 with severe FH demonstrating low specificity in this population [16], and rises with exercise [17].

Assessment with coronary CT angiography (CCTA) provides incremental prognostic value over traditional risk stratification models, though not in direct comparison to the CCS [18]. CCTA incorporates an assessment of prognostic risk factors, e.g. non-calcific plaque and high-risk plaque features (HRP), which are unappreciable with CCS [19, 20]. CCTA may therefore identify patients at increased cardiovascular disease (CVD) risk, which may impact lipid targets and treatment options [21, 22].

Innovative technologies enable extraction of further, sensitive CVD risk data from routine CCTA, including the pericoronary fat attenuation index (FAI). Inflammation drives atherosclerosis [23], and FAI is a novel, artificial intelligence (AI) derived biomarker of coronary inflammation. Studies have demonstrated that FAI enhances existing CVD risk stratification above state-of-the-art models [24, 25]. FAI predicted risk correlates near perfectly with observed risk across a varied cohort of patients, including those with non-obstructive CAD [24]. Recent studies demonstrate that lesion-specific pericoronary FAI enhances the predictive accuracy for MACE when added to the CCS [26].

FAI is not a static value but is modifiable with treatment, including statin therapy [24, 27, 28]. FAI may therefore provide insight into patients who have not responded to treatment and may benefit from an escalation in therapy or identify poor treatment adherence.

This exploratory pilot study aimed to: (i) quantify and compare subclinical atherosclerosis burden in real-world lipid clinic patients assessed with a CCS vs. CCTA; (ii) survey hypothetical clinician response to CCTA versus CCS findings; and (iii) describe the pericoronary FAI biomarker of inflammation in this higher cardiovascular risk cohort.

## Materials and methods

### Patient cohort

We conducted a retrospective review of consecutive cases in our institution’s lipid clinic who had undergone both CCS and CCTA imaging. Patients were referred for imaging at the treating Consultant Lipidologist’s discretion. Our institution is an acute medium-sized Trust with a catchment population of approximately 500,000 and the lipid clinic reviews an average of 100 patients per month.

**Fig. 1:**
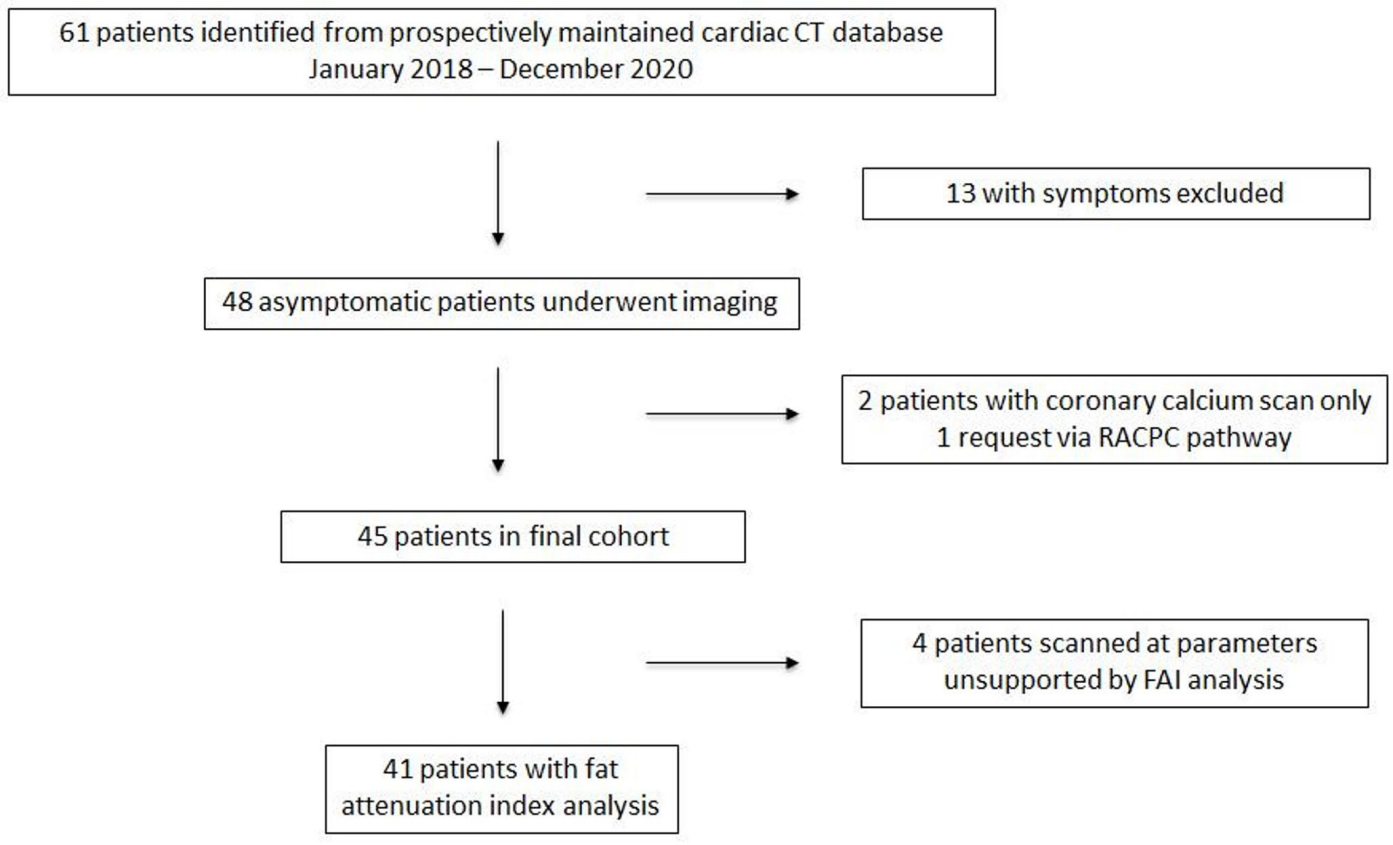
Study flowchart (RACPC = rapid access chest pain clinic, FAI = fat attenuation index)

Patients were identified from a prospectively maintained database of all lipid clinic referrals to CT and cross-referenced with a targeted search of CT request electronic records. Exclusions included symptomatic patients (where a CCTA was indicated in line with UK guidance [29]), patients without both a CCS and CCTA, and if imaging was requested via a non-lipid clinic pathway. For FAI sub-analysis, scans were excluded if acquired with unsupported parameters, e.g. scans acquired at 90 or 110 kV (Fig. 1).

Electronic patient record (EPR) data were collected as accurate at the time of imaging. This included demographics, primary lipid diagnosis, medications, cardiovascular risk factors, family history of premature cardiovascular disease (defined as MACE aged < 60 in 1 st degree relative or < 50 in 2nd degree relative), and blood results (where available), including: full baseline lipid profile (or estimated baseline from on-treatment levels), current glycated haemoglobin (HbA1c), and lipoprotein a (Lp[a]). FH was diagnosed in the presence of a definite FH causing pathogenic variant or in patients with clinical FH (as defined by the Simon Broome criteria) and the presence of a variance of unknown significance with as yet unconfirmed pathogenicity. Polygenic hypercholesterolaemia (PH) was diagnosed by the polygenic risk score in the majority of patients.

MACE rate (composite of death, MI, coronary revascularisation, stroke, and hospitalisation for heart failure) was recorded.

### Cardiac imaging

All imaging was acquired with a 128-detector CT machine (64-detector with flying-focal spot, Siemens Edge, Siemens Healthineers, Erlangen). For all cases, a routine clinical report was produced by a Consultant Radiologist with ≥ 5 years’ experience in cardiac CT.

*CCS*:

A baseline CCS was acquired and reported using the standardised Agatston protocol [30]. The Agatston score was classified as 0 (no disease), 1–10 (minimal disease), 11–100 (mild disease), 101–400 (moderate disease) and >400 (severe disease), as per CAC-DRS [31]. Reports combined Agatston score with an individualised Multi-Ethnic Study of Atherosclerosis (MESA) estimated ‘coronary age’[32].

*CCTA*:

CCTA acquisition parameters are described in the supplementary materials. CCTA studies were read as part of routine clinical practice using Syngo.via post-processing software (Siemens Healthineers, Erlangen). Stenoses were reported as per the internationally agreed Coronary Artery Disease—Reporting and Data System (CAD-RADS™ 1.0[33]). This assigns an ordinal number related to the degree of luminal stenosis in cross-sectional analysis (0 = no stenosis, 1 = 1–24% [minimal], 2 = 25–49% [mild], 3 = 50–69% [moderate], 4 = 70–99% [severe], 5 = 100% [occluded]). A modifier for HRP was added if ≥ 2 were present on CCTA.

*Coronary Artery Perivascular FAI*:

All CCTA studies were analysed using the CaRi-Heart^®^ device (Caristo Diagnostics) to calculate FAI and FAI-score, as previously described and validated [24, 25, 34, 35]. All scans were anonymised, and the reader was blinded to demographics and time of the scan. Age, sex and major cardiovascular comorbidities were then added to provide a per-vessel FAI-score alongside the percentile of coronary inflammation matched for age and gender. FAI-score percentile category was defined as high if >75th percentile for age- and sex-matched controls, intermediate if 50–75th percentile and low if < 50th percentile. Further information is available in the supplementary materials.

### Lipidologist survey

Consultant Lipidologists from across the UK were invited to participate in an online survey assessing the impact of CT results on hypothetical lipid clinic patient management. Clinicians contributing were based in Trusts across Bristol, Shropshire, Sussex, Salisbury, Plymouth and Newcastle.

All participating clinicians were sent background educational material on the study and cardiac CT (see supplementary material) to mitigate against variation in prior level of exposure to cardiac CT. Respondents could move forwards and backwards within the survey, or save and return to complete at serial intervals. To reflect real-world practice, clinicians were given no prior direction on which lipid guidelines to base treatment targets and management.

Clinical case summaries (vignettes) were anonymised and converted into an online survey. Data was revealed in a step-wise fashion for each case: (i) clinical vignette (summarising EPR), (ii) CCS, (iii) CCTA assessment. At each step, clinicians were asked for their lipid target and whether any other management was indicated. The percentage of respondents altering their lipid target was recorded, broken down by CCS, CCTA, and sub-divided by severity of CAD (as assessed by each imaging modality).

### Statistical analysis

Statistical analysis was performed with SPSS v.21 (Armonk, NY, USA: IBM Corp). Categorical data are presented as frequency (percentage), and continuous data as mean ± standard deviation or median with interquartile range (IQR) for non-parametric data. The re-classification of coronary disease presence and severity was tested with the McNemar and marginal homogeneity test respectively. CCS severity was compared with FAI-score percentile group using the Chi-squared test.

Survey analysis was undertaken on a per-patient, per-rater basis to assess the impact of sequential clinical information made available on treatment targets and clinical management. Inter-observer agreement was assessed with multi-rater Fleiss’ κ statistic. Agreement was analysed after baseline clinical vignette only, and then after each incremental piece of CT information was unblinded. Fleiss’ κ was categorised as poor (κ⩽0.20), fair (0.20 < κ ⩽0.40), moderate (0.40 < κ ⩽0.60), good (0.60 < κ ⩽0.80) and excellent (0.80 < κ ⩽1.00). Change in LDL target was measured with the McNemar test.

For MACE outcome analysis, CT date was considered the start of follow-up and the study cut-off point for censoring defined as at event or February 28th 2024. Significance was defined as two-tailed *p* < 0.05 throughout.

### Ethics

This study was approved as a retrospective service evaluation project by our institution’s Trust Audit Committee, waiving the requirement for formal written consent in line with the Health Research Author decision tool [36]. FAI analysis was performed under Arm 4 of the Oxford Risk Factors and Non-invasive imaging study (ORFAN), which has received approval by South Central – Oxford C Research Ethics Committee (REC reference 15/SC/0545).

## Results

61 potentially eligible patients were identified, with patients excluded for symptoms (13), lack of CCTA (2) and for a referral via a non-lipid clinic pathway (1). The final cohort therefore included 45 asymptomatic patients (imaged January 2018-December 2020), with 49% female (22) and mean age 54 (± 9) years (see Fig. 1). Table 1 provides baseline patient characteristics.

### Coronary calcium scan (CCS) vs. coronary CT angiography (CCTA)

Coronary artery calcification (CAC) of any degree was observed in 64% (29/45) of patients. The overall CCS breakdown across the cohort is provided in Table 2, sub-divided by lipid diagnosis.

**Fig. 2:**
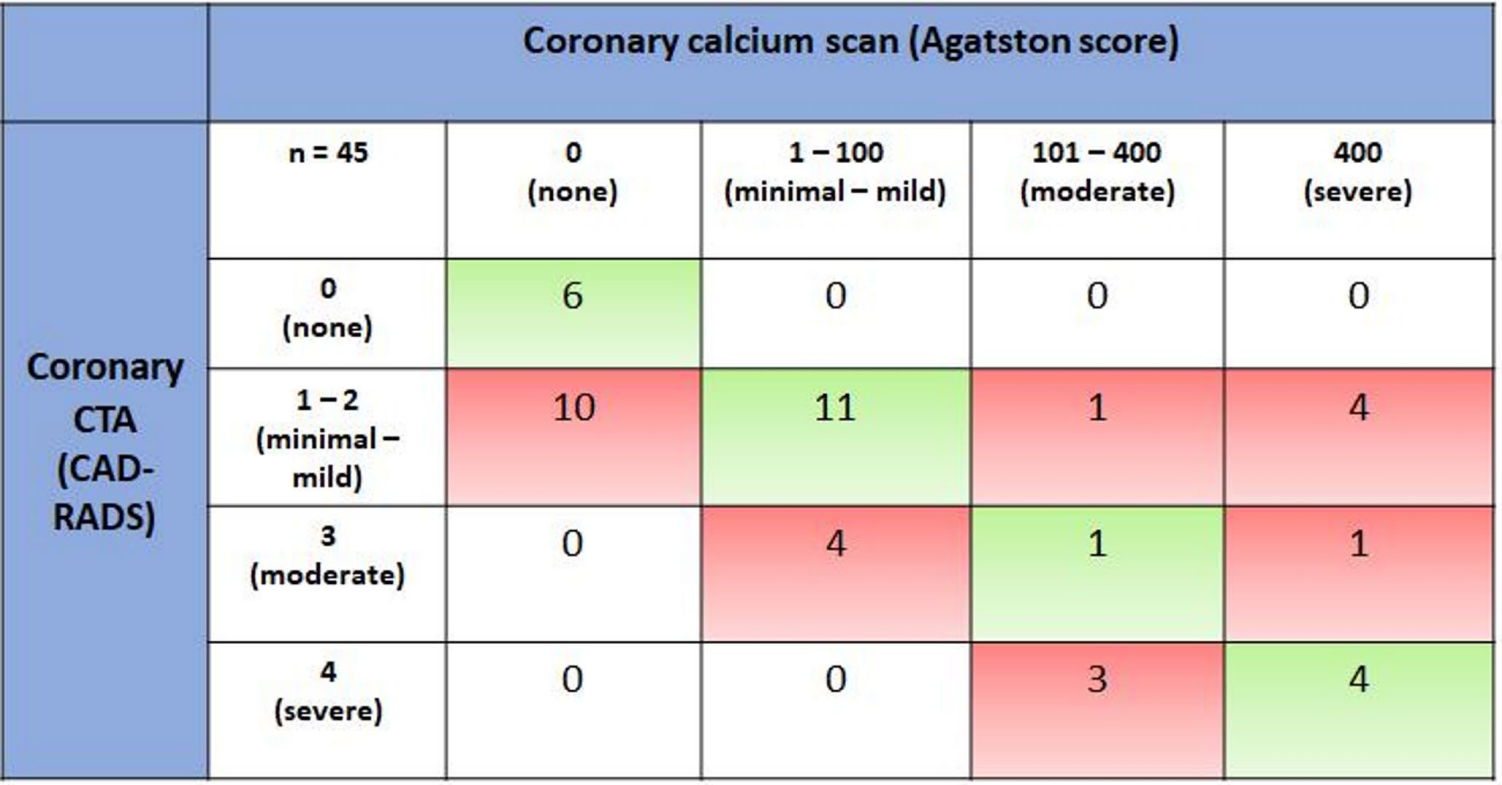
Comparison of coronary calcium score defined CAD severity category with CCTA (assessed with CAD-RADS). Green cells demonstrate agreement between imaging modalities and red cells denote change in CAD grading

CAD prevalence increased to 87% (39/45) of patients when assessed with CCTA, which re-classified CAD presence vs. CCS in 22% (10/45) of patients (*p* = 0.002). CAD severity grade was re-classified by CCTA vs. CCS in 62% (28/45) of patients (*p* = 0.005). CCTA increased CAD severity grade in 40% (18/45), and downgraded in 22% (10/45, see Fig. 2). The escalation in CAD severity was predominantly driven by patients moving from CCS 0 to CAD-RADS 1 (50% [9/18] of patients with an increase in CAD grade). All 4 cases (100%) where a CCS of > 400 was downgraded to a CAD RADS of 1–2 were taking high-intensity statin vs. 3/5 (60%) of those with CCS > 400 and a CAD RADS 3–4.

Comparing CCTA-defined CAD severity with CCS assessment, no CAD was observed in 13% (6) vs. 36% (16), minimal in 40% (18) vs. 11% (5), mild in 18% (8) vs. 22% (10), moderate in 13% (6) vs. 5 (11%) and severe CAD in 16% (7) vs. 20% (9), as demonstrated in Fig. 2.

HRP features were observed in 20% (9/45) of patients. Of these, 56% (5/9) had a CCS < 100 (mild) or lower, including 1 patient with a score of 0. 56% (5/9) had a CCTA-defined CAD-RADS category ≤ 2 (≤ mild).

**Table 1:** Baseline patient demographics

Demographic	(*n* = 45)
Age (years)	54 ± 9
Female gender (%)	22 (49%)
Hypertension	7 (16%)
Diabetes mellitus	1 (2%)
Family history of premature CVD	27 (60%)
Lipid diagnosis	
Familial hypercholesterolaemia	22 (49%)
Polygenic hypercholesterolaemia	13 (29%)
Familial combined hyperlipidaemia	5 (11%)
Mixed/metabolic hyperlipidaemia	5 (11%)
Smoking	
Current	5 (11%)
Ex-smoker	7 (16%)
Never smoker	26 (57%)
Unknown	7 (16%)
Obesity (BMI ≥30 kg/m^2^)	8 (18%)
Lipid treatment	
Statin	34 (76%)
High-intensity statin	30 (67%)
Ezetimibe	17 (38%)
Bile acid sequestrant	2 (4%)
PCSK9i	2 (4%)
Baseline blood tests	
TC [mg/dL]	317 (±70)
LDL [mg/dL]	232 (±81)
HDL [mg/dL]	54 (±15)
Trig. [mg/dL]	213 (±151)
Lp(a) [mg/L]	249 (IQR 91–1155)
HbA1c [mmol/mol]	38 (±6.2)

CVD = cardiovascular disease, PCSK9i = pro-protein convertase subtilisin/kexin type 9 inhibitor, TC = total cholesterol, LDL = low-density lipoprotein, HDL = high-density lipoprotein, Trig. = triglycerides, Lp(a) = lipoprotein (a), HbA1c = glycated haemoglobin, IQR = interquartile range

**Table 2:** Imaging findings across modalities assessed sub-divided by lipid diagnosis

Lipid Diagnosis	Agatston Score (%[*n*])					CAD-RADS (%[*n*])					HRP (%[*n*])	FAI (%[*n*])		
	None	10-Jan	11–100	101–400	>400	0	1	2	3	4		Low	inter.	High
All	36 [16]	11 [5]	22 [10]	11 [5]	20 [9]	13 [6]	40 [18]	18 [8]	13 [6]	16 [7]	20 [9]	44 [18]	36 [15]	20 [8]
FH (22)	27 [6]	14 [3]	23 [5]	14 [3]	23 [5]	14 [3]	41 [9]	18 [4]	5 [1]	23 [5]	18 [4]	38 [8]	33 [7]	29 [6]
PH (13)	31 [4]	16 [2]	23 [3]	16 [2]	16 [2]	0 [0]	38.5 [5]	23 [3]	23 [3]	16 [2]	16 [2]	46 [5]	46 [5]	9 [1]
FCH (5)	60 [3]	0 [0]	20 [1]	0 [0]	20 [1]	40 [2]	20 [1]	0 [0]	40 [2]	0 [0]	40 [2]	40 [2]	60 [3]	0 [0]
Mixed/metabolic (5)	60 [3]	0 [0]	20 [1]	0 [0]	20 [1]	20 [1]	60 [3]	20 [1]	0 [0]	0 [0]	20 [1]	75 [3]	0 [0]	25 [1]

FH = familial hypercholesterolaemia, PH = polygenic hypercholesterolaemia, FCH = familial combined hyperlipidaemia, CAD-RADS = Coronary Artery Disease—Reporting and Data System, HRP = high-risk plaque, FAI = fat attenuation index, Inter. = intermediate

### Lipidologist survey

The survey was completed by 7 Consultants from 6 institutions, 4 (67%) tertiary care or university teaching hospitals and 2 (33%) district general hospitals. With 7 clinicians reviewing 45 cases, this enabled 315 opportunities to alter LDL target or management for each layered, sequential CT data-point.

*Impact on treatment targets*

The median hypothetical LDL target selected based on the clinical vignette alone was 101 mg/dL (IQR 77–120). This reduced to 89 mg/dL (IQR 77–120) after the CCS was revealed, and 77 mg/dL (IQR 70–116) after CCTA-defined CAD-RADS score was unblinded.

Across all 45 cases reviewed, the level of agreement on LDL target based on clinical vignette alone was categorised as poor (κ = 0.20). This reduced with CCS (κ = 0.15) and CAD-RADS (κ = 0.11).

Overall, the hypothetical LDL target selected was altered by the CCS in 12% (38/315), and in a further 19% (60/315) when CCTA findings were unblinded, which was statistically significant (χ^2^ 57.0, *p* < 0.005). The frequency of LDL target adjustment with CCS and CCTA, broken down by severity category for each modality, is provided in Table S1.

When comparing patients with FH versus a non-FH lipid diagnosis, the CCS changed management in 12% (19/154) with FH versus 12% (19/161) non-FH, and the CCTA in 22% (34/154) with FH versus 29% (46/161) non-FH.

### Associated management

Respondents were asked for any other management required after each layer of clinical data was unblinded. There was an increase in referrals for cardiology advice or review with escalating CCS and CAD-RADS severity, alongside an increase in newly prescribed aspirin (Table S2).

### Sub-analysis: CCS vs. CCTA with FAI

**Fig. 3:**
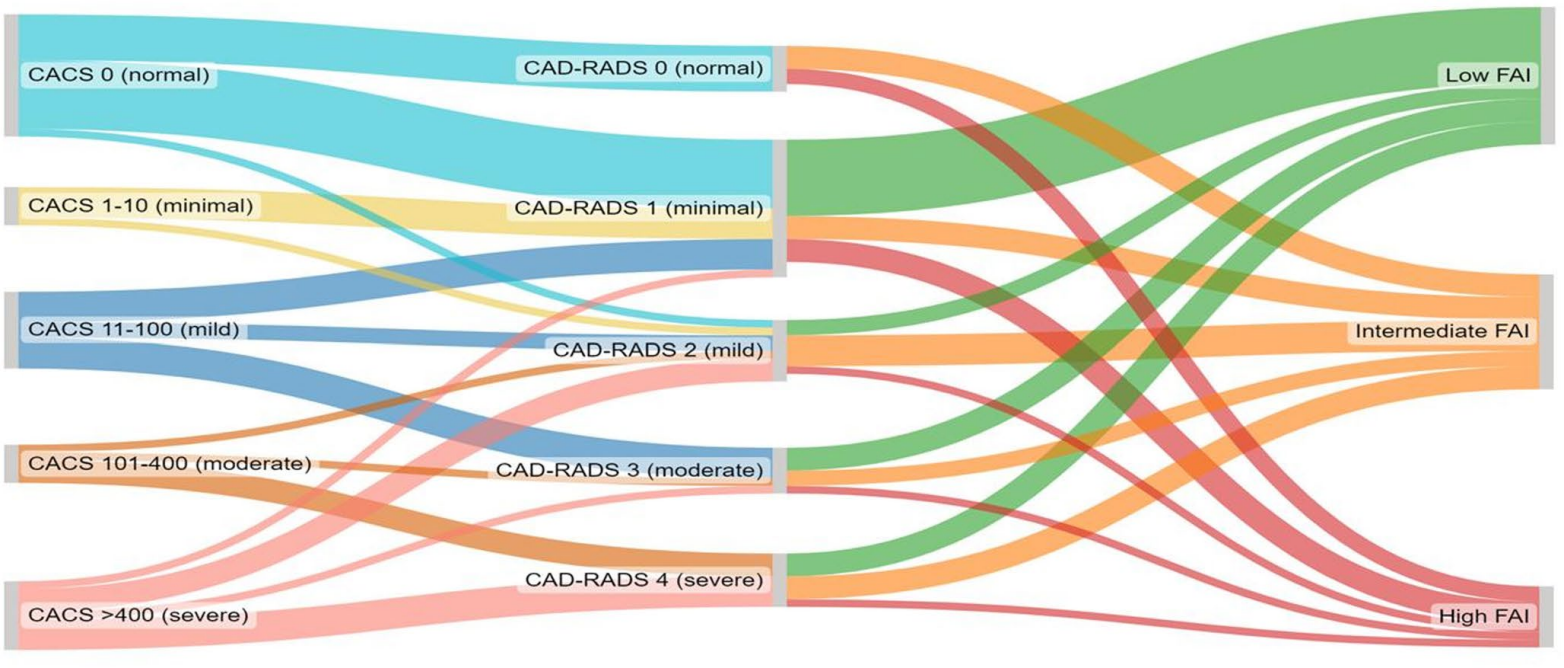
Re-classification of coronary artery disease severity when assessed by CCS vs. CCTA vs. FAI. (CACS = coronary artery calcium score, CAD-RADS = Coronary Artery Disease—Reporting and Data System, FAI = fat attenuation index, HRP = high-risk plaque)

**Fig. 4:**
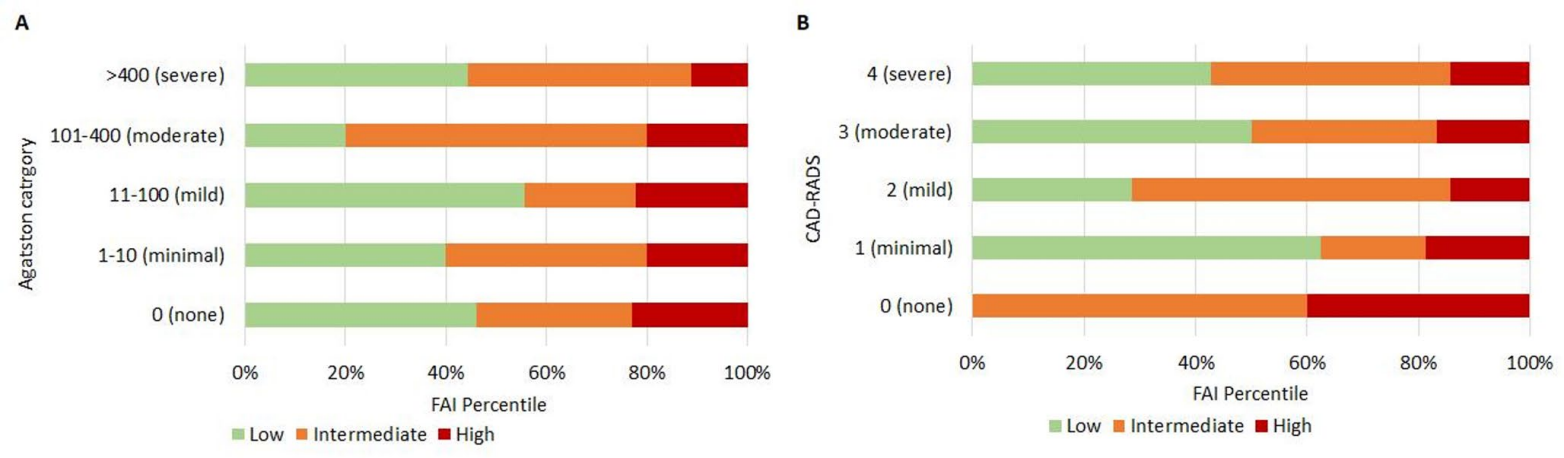
Severity of CAD as defined by coronary calcium score (Agatston category), panel A) and CT coronary angiography defined CAD-RADS category (panel B) broken down by presence of low (< 50%), intermediate (50–75%) or high (> 75%) FAI-score (any vessel). (FAI = fat attenuation index, CAD-RADS = Coronary Artery Disease—Reporting and Data System)

**Fig. 5:**
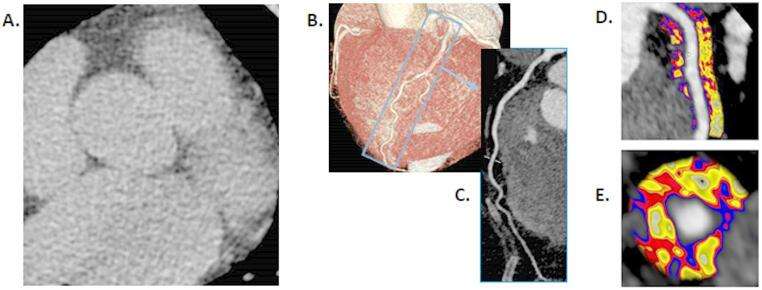
A case example of a 67-year-old female patient with mixed dyslipidaemia. Panel A demonstrates a single axial slice of the baseline coronary calcium score, which is normal with an Agatston score of 0; Panel B demonstrates a curved multiplanar reconstruction of this patient’s CT coronary angiogram; Panel C provides a reconstruction of the left anterior descending artery (LAD) from this CT, which is normal with no evidence of plaque; and Panels D and E demonstrates fat attenuation index (FAI) analysis of the same LAD from panel C, which is significantly abnormal (FAI − 69.4HU and FAI-score in the 87th percentile for age and gender) shown visually by the red and blue colouring in the perivascular space

FAI analysis was available in 91% (41/45) of patients. The FAI-score further re-stratified risk relative to both the CCS and CCTA (Fig. 3). High FAI-score was seen across all severity groups of CCS and CAD-RADS scores (Fig. 4). This included in 22% (6/27) of patients with none to mild calcification on CCS and 21% (6/28) of patients with none to mild CAD on CCTA. An example case is presented in Fig. 5. The proportion with high FAI-score was greater in CAD-RADS 0 (33% [2/6]) vs. CAD-RADS 4 (14% [1/7), whilst CCS grade was not associated with the maximum FAI-score percentile risk category observed (*X*^2^ = 2.95, *p* = 0.94).

HRP was observed in 13% (1/8) of patients with high FAI-score, 20% (3/15) with intermediate and 22% (4/18) with low. There was no association between HRP & FAI-score percentile category (*X*^2^ = 0.40, *p* = 0.70), with 4/8 (50%) having HRP and a low FAI-score percentile, 3 (38%) intermediate and 1 (12%) high.

High FAI-score was observed in 17% (6/35) of patients on treatment versus 33% (2/6) not at the time of cardiac imaging. This included patients with a CCS ranging from 0 to severe (> 400), and a CAD-RADS of minimal (1) to severe (4). There was no association between presence of a statin (*p* = 0.41) or high-intensity statin (*p* = 0.85) and FAI percentile category.

### Outcomes

After a median follow-up of 53 months (IQR 50–56), total MACE rate was 9% (4/45). Across the 4 cases, CCS ranged from 18 (mild) to 487 (severe), CAD RADS from 2 V (modifier for HRP) to 4bV, whilst all patients with coronary events had intermediate or high FAI.

## Discussion

This exploratory pilot study demonstrates the potential for re-stratification of CAD presence and severity using CCTA in a high cardiovascular risk population when compared to the current standard of care, the CCS, alongside its potential impact on clinical management. This is also the first study to measure FAI in a dedicated lipid clinic cohort.

Whilst dyslipidaemia promotes premature atherosclerosis and associated MACE, this risk is heterogeneous, even within the same sub-group of dyslipidaemia. For example, the severity and clinical expression of FH is known to vary even within a family sharing the same pathogenic mutation [37]. Risk factors beyond prolonged exposure to high levels of LDL play a role. Cardiac CT is increasingly recognised as offering a simple quantification of CAD that integrates the interplay of these cumulative risk factors into a single, reproducible and actionable measure of risk [38]. In keeping with this, contemporary AI-enabled quantitative CCTA now allows automated plaque burden and composition measurements that have been linked to outcomes and, importantly, show sex-specific prognostic differences [39].

CCS is currently first-line for asymptomatic patients in guidelines. However, CCS misses non-calcific plaque, lower volume calcific plaque below the threshold for reporting in the Agatston protocol, HRP features and cannot grade stenosis. CCS therefore has no role in the assessment of symptomatic patients, in whom CCTA is first-line [11, 29, 40]. Even for patients with zero CAC, the presence of non-calcified plaque, HRP features and stenosis on CCTA is prognostically superior to CAC testing and has clinical implications for the timing and intensity of preventive therapies [41, 42]. In addition, a recent meta-analysis indicates that CAC density (distinct from score or volume) is inversely associated with cardiovascular risk after adjustment for traditional risk factors and CAC burden, suggesting that denser calcification may reflect more stable plaque biology and should be interpreted alongside (not instead of) total CAC [43].

The SCOT-HEART study tested CCTA as the first-line investigation of stable chest pain versus standard of care [44]. This demonstrated the prognostic benefit of a CCTA strategy, and the largest proportional reduction in non-fatal MI or death from CAD was observed in patients with non-anginal chest pain [45]. For many individuals this may represent incidental identification of CAD that did not account for their non-cardiac symptoms.

In our study of asymptomatic lipid clinic patients, CAC (of any degree) was observed in 64% (29/45) of patients. A subsequent CCTA re-classified CAD severity in a significant proportion of patients (62%), including newly diagnosing CAD in 22% (10/45) of patients with zero CAC. This suggests a potential underestimation of CAD risk when using CCS alone, which may have important clinical implications for risk stratification and management in lipid clinics. Similar findings were observed in the PROMISE and SCOT-HEART studies, where 16% and 17% of patients respectively, had evidence of coronary atherosclerosis on CCTA despite a CCS of zero [41, 42]. The slightly higher proportion in our study (22%) may reflect the higher cardiovascular risk cohort seen in the lipid clinic. Indeed, high Lp(a) was recently shown to accelerate plaque burden and increase both non-calcific plaque and FAI [46]. Moreover, findings from the CONFIRM2 Registry show that quantitative plaque phenotypes carry a stronger relative association with MACE in women than in men, reinforcing the need to look beyond CCS alone when evaluating high-risk female patients [39].

HRP features were identified in 20% of the cohort, present across all lipid diagnoses. HRP was observed in 23% of all patients with a CCS in the zero to mild range (< 100) – patients who would otherwise have been considered lower risk. Identification of subclinical CAD infers support for informed decision-making regarding the use and intensity of preventative medications and therapeutic lifestyle changes by patients. Its impact on outcomes does though require assessment in a prospective, longer-term study. This aligns with recent work showing that premature CAD is characterised by a higher prevalence of non-calcified plaque and multiple HRP features on CCTA compared with incidental plaques in matched controls, highlighting the aggressive, composition-heavy phenotype in younger patients [47].

Though the absolute numbers were low, the presence of a high-intensity statin in all patients with a CCS > 400 where CAD RADS was 1–2 vs. 60% of those where CAD RADS was 3–4 may highlight the need to be cautious in over-interpreting the CCS in patients taking high-intensity statins.

As the first study to quantify the impact of cardiac imaging on the hypothetical management of a real-world lipid clinic cohort, we observed a lowering of the LDL target selected with the incremental data provided by both a CCS and CCTA. International guidelines advise that in asymptomatic individuals with higher CVD risk (including with Agatston score >100 AU), the use of CCTA assessment may reclassify an individual to a higher risk category [7, 11, 48]. We demonstrated a reduction in the hypothetical median LDL target selected with CCTA, even after clinicians had been unblinded to the CCS. Indeed, even in patients with severe CAC (i.e. CCS >400 AU), there was a further lowering of median target LDL with CCTA results.

In the context of the study size and hypothetical nature of the management selected, caution should be applied in over-interpreting the potential clinical impact of CCTA, with study findings serving as hypothesis-generating. Indeed, whilst previous randomised trial data have demonstrated the ability for CCS to significantly re-stratify CVD risk [49], to date this has not been demonstrated with CCTA in asymptomatic patients. In the CONFIRM registry CCTA did not significantly re-stratify risk above CCS [49]. However, the imaging landscape and CCTA techniques continue to evole. The multicentre CONFIRM2 Registry reported significant, sex-differential prognostic associations for quantitative plaque features, suggesting that modern techniques may provide risk information not captured by legacy analyses [39]. This warrants prospective validation in higher-risk, asymptomatic cohorts such as lipid clinic populations.

Increasing focus is placed on recognising patients with proven atherosclerosis as at higher risk of future MACE. European guidelines recommend both the consideration of cardiac imaging to support enhanced risk stratification, and more aggressive targets and treatment options when ASCVD is present. However, these recommendations differ across UK, European and American lipid guidance [50]. In addition, the interpretation of primary versus secondary prevention guidelines in the setting of an asymptomatic patient with proven atherosclerosis but no history of established ischaemic or cerebrovascular disease may vary amongst clinicians. As such, there was significant variation in management selected, demonstrated by the low level of agreement on LDL targets amongst respondents. Given the sex-specific quantitative plaque data and the protective signal of higher CAC density, harmonising guidance on how to integrate plaque composition and calcification density into LDL-target selection may reduce practice variability [43].

Of note, agreement was graded as poor even after the clinical vignette alone. There are a variety of potential factors that may have influenced this finding, including insufficient prior respondent exposure to CTCA results or the potential for ambiguous case vignettes. A key consideration however is whether variability reflects the differing use of guidelines and varying interpretations of cardiovascular risk stratification. If agreement amongst experts in this field is poor, this suggests a need for greater consensus across national and international guidance to improve consistency in patient management. However, the validity of these insights is limited by the hypothetical nature of the survey responses and the small number of respondents, which may not represent broader clinical practice.

The level of clinician agreement also deteriorated with each layer of cardiac imaging. We attempted to improve consistency in the interpretation of CT findings with the provision of educational materials in advance of survey completion (supplementary materials). However, the reduction in agreement may still reflect both variation in clinician’s prior exposure to cardiac imaging and interpretation of results in the context of the differing guidelines available. There is also debate as to what constitutes sufficient plaque within the coronary tree, or indeed across multiple vascular beds, to warrant a lowering of LDL targets and escalation in treatment. Recently published age- and sex-specific nomograms for quantitative plaque volumes provide reference percentiles derived from a large, international cohort and may help standardise interpretation of plaque burden [51].

The variation in baseline LDL target set may impede interpretation of clinician response to subsequent cardiac imaging. Allowing for this and despite the reduction in level of agreement across respondents, there remained a statistically significant decrease in LDL target set with CCTA results even after clinicians had been provided with a CCS. The increasing use of non-invasive cardiac imaging appears to offer an opportunity to differentiate high and very high cardiovascular risk in populations such as those seen in the lipid clinic. Demonstrable evidence of CAD may also improve patient adherence to treatment. These hypotheses require testing in a prospective, randomised study.

As demonstrated in this study, however, there may be a down-stream impact on other services. With increasing burden of CAD detected there were rising cardiology referrals considered. Whilst this may be appropriate for some cases, increased use of cardiac imaging would require a broader discussion around the appropriate investigation and management of asymptomatic CAD, particularly in light of recent evidence [52].

Whilst studies have assessed the role of cardiac imaging and burden of subclinical atherosclerosis in FH, there has been less focus on other dyslipidaemias associated with heightened risk of MACE. Our study observed the potential for cardiac imaging to play a role in the re-stratification of cardiovascular risk across conditions seen in the lipid clinic. Whether this translates into changes in clinical outcomes was beyond the scope of this study.

Recent work has suggested a low short-term MACE risk of heterozygous FH patients with a CCS of 0 [53]. However, this study was limited by a relatively short follow-up period (particularly relevant given the younger age of the population studied) and overall low event rates. Indeed, in the REFERCHOL study, patients with heterozygous FH and no prior history of CVD had a MACE rate of 9.4% at 5 years [53]. This compares with our findings of 9% at just over 4 years, and 75% (3/4) of these had a CCS graded mild or moderate. Further data on longer-term risk for patients assessed with CCTA is required given this patient group’s elevated lifetime risk for MACE. However, as suggested in a clinical practice statement from the American Society for Preventive Cardiology, “as we await studies, a judicious approach to use of CCTA in asymptomatic populations is to target high-risk populations that are currently missed by traditional ASCVD risk factor scoring” [54].

This study demonstrates the potential for further re-stratification of cardiovascular risk in this cohort with layered FAI analysis (available from CCTA). FAI-score is a sensitive biomarker of coronary inflammation known to enhance cardiovascular risk prediction above current standard of care [24], though it should be noted FAI remains an investigational tool in this context requiring prospective validation. In a recent study FAI-score was shown to be an accurate predictor of both cardiac mortality and MACE, independent of other cardiovascular risk factors and the presence or extent of CAD [25]. Indeed, the predicted risk correlated almost perfectly with observed risk across a diverse cohort of patients with varying pretest probabilities, including patients with minimal or non-obstructive CAD (i.e. cases without typical angina from stenotic epicardial disease).

In our study, FAI-score re-stratified the cohort relative to both CCS and CCTA across all lipid diagnoses (Table 2). Interestingly, high FAI-score was observed in 22% of patients with none to mild calcification on CCS (< 100 AU) and 21% of patients with none to mild CAD when defined by stenosis on CCTA (CAD-RADS 0–2). The proportion with a high FAI-score was greater in patients with a CCTA demonstrating no plaque than those with a severe stenosis. It may be that the incremental risk stratification provided by FAI analysis can play a role in the treatment decisions for patients with a lower burden of a disease on baseline imaging. However, the assessment of this is limited by the sample size of the study and requires further work including longer-term outcome data.

FAI is modifiable with treatment. In a sub-analysis of the CRISP-CT study, FAI did not retain its significant association with subsequent MACE in patients who received initial treatment with statins or aspirin after CCTA [24]. These findings were confirmed by other groups [27] who found the perivascular FAI to be a dynamic tool for monitoring response to statin treatment, demonstrating a significant reduction in the pericoronary FAI around non-calcified and mixed plaques but not around calcified plaques. FAI may therefore identify patients with persistent higher risk not responding to treatment, or those with poor adherence.

In our study, high FAI-score was observed in 17% of patients on treatment compared with 33% of patients on no treatment at the time of their imaging. We did not find an association between either any statin or a high-intensity statin and FAI, whilst the low number of patients on PCSK9-inhibitors (2) at the time of CT prevented analysis. The impact of treatment dictated by FAI-score requires further interrogation in a larger cohort tracking MACE.

### Limitations

This exploratory pilot study is limited by its single-centre and retrospective design, limiting the ability to control for confounders. The modest sample size and heterogeneity of the population limit interpretation of findings, particularly sub-analyses, though offer exploratory, hypothesis-generating data. The patient cohort reflects clinician’s discretion as to which patients cardiac imaging were considered in, rather than a random process or systematically applied to all patients, leaving the potential for selection bias. This, combined with the modest sample size and single-centre design may limit the representativeness of the cohort and generalisability of our findings to differing lipid clinic populations.

Allowing for this though, study results demonstrate that the current methods of estimating risk are heterogeneous. The number of raters was modest, and the assessment of impact on management was based on hypothetical decision-making rather than the prospective decisions taken in each case, which may not represent broader clinical practice. However, the use of real-world consecutive clinical cases referred for cardiac CT enhances its application to the lipid clinic and reflects current practice. Further, the involvement of clinicians from a variety of institutions across the UK demonstrates a sampling of clinical practice and adds weight to findings.

The relatively large number of sequential responses required within the survey may have risked a degree of response-fatigue. This was mitigated by allowing respondents to pause, exit and return at the same point, completing the survey over multiple sittings at a time to suit them. A potential lack of clinician routine exposure to CCTA prior to participation results may have influenced responses, though this was partially addressed by the provision of standardised supplemental educational material to all respondents and reflects current practice. Further research will need to consider the health economic and longer-term clinical impact of CCTA +/- FAI versus standard of care in larger, prospective multi-centre cohort studies.

## Conclusion

In this exploratory pilot study, CCTA re-stratified CAD presence and severity vs. CCS in a heterogeneous lipid clinic cohort. Clinician survey results demonstrate the potential for CCTA to change LDL targets and management, even after reviewing CCS findings. Variation in clinician agreement may reflect a lack of consensus across lipid guidelines, whilst the interpretation of clinical impact is limited by the hypothetical nature of responses. Layered FAI analysis detected patients at risk of future MACE regardless of CCS and CCTA findings, including patients without evidence of overt CAD. Larger, prospective studies are required to further assess.

## Supplementary Information

Below is the link to the electronic supplementary material.

Supplementary Material 1

## Data Availability

Data is provided within the manuscript or supplementary information files. Raw data is available upon request.
