# Supplementary material for: CT coronary angiography in the lipid clinic: a pilot study and lipidologist survey
Source: Int J Cardiovasc Imaging. 2025 Oct 9;41(11):2205–17. doi: 10.1007/s10554-025-03526-3 (PMC12628484; doi:10.1007/s10554-025-03526-3)
Supplement: Supplementary file 1 — Supplementary Material 1 [file 10554_2025_3526_MOESM1_ESM.docx]

**Supplementary Data**

*CT coronary angiography*

CTCA acquisition parameters were as follows: test bolus technique (12 ml Niopam 340 at 6 ml/s), coronary CTA (60 ml Niopam 340 at 6 ml/s), section thickness of 0.6mm, a pitch sequential scan feed of 34.5 mm, rotation time 0.28 seconds, and a tube voltage reference of 100 kVp with automated voltage modulation and 220 reference mAs with automated tube current modulation. Default CTCA reconstructions were: cardiac field of view of 0.6 mm coronary vascular reconstruction kernel (I26f, Siemens Healthineers), 0.6 mm sharp vascular reconstruction kernel (I46f, Siemens Healthineers), and 0.6 mm raw transverse reconstructions without automated smoothing between steps (Truestack I30f, Siemens Healthineers).

*Pericoronary fat attenuation index*

For use in clinical practice, FAI needs to be standardised for a number of technical (e.g. CT tube voltage) and clinical (e.g. age, gender) factors. These have been incorporated into a CE-marked medical device, CaRi-Heart^®^31. FAI was measured in the proximal portion of all three major epicardial vessel (left anterior descending [LAD], right coronary artery [RCA], and left circumflex [LCx]), and defined as the weighted mean attenuation of all adipose tissue–containing voxels (−190 to −30 Hounsfield units [HU]) located within a radial distance from the outer vessel wall equal to the diameter of the respective vessel.

**Table S1.** Frequency of change in LDL target made by clinicians with CCS vs CTCA, broken down by severity category for each modality. 7 clinicians individually reviewed 45 cases, thus there were n=315 potential occasions for a change in LDL target.

**Frequency of LDL target adjustment**

**CCS** (n=315) **CTCA (CAD-RADS)** (n=315)

n % n %

0 (normal) 0/112 0 0 (normal) 0/42 0

1 – 10 (minimal) 2/35 6 1 (minimal) 4/119 3

1V 5/7 71

11 – 100 (mild) 6/70 9 2 (mild) 1/14 7

2V 18/42 44

101 – 400 (moderate) 9/35 26 3 (moderate) 1/14 7

3V 13/28 48

>400 (severe) 20/63 32 4 (severe) 10/28 36

4V 8/21 38

**Table S2.** Additional management selected after each of the clinical vignette, CCS and CAD-RADS data were unblended to the responders, sub-divided by severity of coronary artery disease.

.

Cardio referral (% [n=number of responders Commence aspirin (% [n=number of responders

changing management]) changing management])

Clinical Vignette

All (n=45) 0 [0] 3 [11]

CCS

0 (n=16) 1 [1] 0 [0]

1 – 10 (n=5) 14 [5] 3 [1]

11 – 100 (n=10) 13 [9] 6 [4]

101 – 400 (n=5) 14 [5] 20 [7]

>400 (n=8 [+1 on aspirin]) 24 [15] 29 [16]

.

CAD-RADS

0 (n=6) 0 [0] 0 [0]

1 (n=17) 0 [0] 0 [0]

1V (n=1) 29 [2] 29 [2]

2 (n=2) 7 [1] 0 [0]

2V (n=6) 43 [18] 48 [20]

3 (n=2) 29 [4] 7 [1]

3V (n=4) 61 [17] 50 [14]

4 (n=4) 71 [20] 32 [9]

4V (n=2 [+1 on aspirin]) 95 [20] 50 [7]

**The Role of CTCA in the lipid clinic**

***Study Background***

Within this case series you will be presented with anonymised clinical vignettes for 45 patients. Initially you will be asked what your lipid target and clinical management would be on the basis of the clinical information only. This clinical information is from the time-point the patient was seen in clinic and the cardiac CT requested.

You will then be presented with the following incremental pieces of relevant information from cardiac CT, and asked for any change to lipid target and management (which should include consideration of adding other therapies, e.g. Aspirin or refer to other speciality) with each layered piece of information:

1. Coronary calcium score (CCS) with “coronary age” (based on The Multi-Ethnic Study of Atherosclerosis (MESA) is a medical research study – N.B., this is not validated in patients <45 years of age)
2. Grading of coronary stenosis based on the nationally approved CAD RADS reporting technique (explained below), which includes a modifier if there are features of vulnerable plaque present (again explained below)

All patients were referred as asymptomatic of coronary artery disease and so any abnormalities identified are considered to represent sub-clinical disease

Whilst we anticipate all physicians will be familiar with the CCS, to assist with interpretation here is a short summary of the CAD RADS system including high-risk plaque features. We recommend this is kept readily available as you work through the patients described in the survey to assist with decision-making.

*CAD-RADS Score:*

The Coronary Artery Disease - Reporting and Data System (CAD-RADS) is a standardised and validated findings communication method and clinical decision aid relevant to coronary CT angiography. Escalating CAD-RADS score correlates to severity of coronary artery disease and individual mortality risk^2^.

A CAD-RADS classification is applied per patient, representing the most severely obstructive coronary artery lesion identified.

Interpretation categories:

Stable chest pain

1. CAD-RADS 0: documented *absence* of coronary artery disease, i.e. 0% maximal coronary stenosis and no plaque
2. CAD-RADS 1: *minimal* non-obstructive coronary artery disease, with 1-24% maximal coronary stenosis, or plaque with no stenosis (positive remodelling)
3. CAD-RADS 2: *mild* non-obstructive coronary artery disease, with 25-49% maximal coronary stenosis = mild stenosis
4. CAD-RADS 3: *moderate* stenosis, with 50-69% maximal coronary stenosis
5. CAD-RADS 4: *severe* stenosis
   1. 4A: 70-99% maximal coronary stenosis
   2. 4B: left main stem >50% stenosis or three-vessel obstructive (≥70% stenosis) disease
6. CAD-RADS 5: total coronary occlusion, i.e. a 100% maximal coronary stenosis
7. CAD-RADS N: non-diagnostic study

If coronary disease identified includes high-risk plaque (described immediately below), a modifier is added to the CAD RADS Score – “V”. E.g., a patient with mild (25-49%) coronary disease and two high-risk plaques will be scored as “*CAD RADS 2V (with two with-risk plaques)*”.

*High-risk plaque features:*

CTCA provides the ability to not only assess for presence of coronary artery disease, but also assess the characteristic of individual coronary plaque identified. High-risk plaque (HRP) features are morphological changes in the composition of coronary plaque (as assessed on a coronary CT) that have been proven to be associated with an increased risk of a future acute coronary syndrome^3,4^. They are generally considered clinically significant when two or more are present. This was robustly validated in a SCOT-HEART sub-study as demonstrated in the graph below^4^, comparing occurrence of coronary heart disease death or non-fatal MI across all patients with and without adverse plaque.


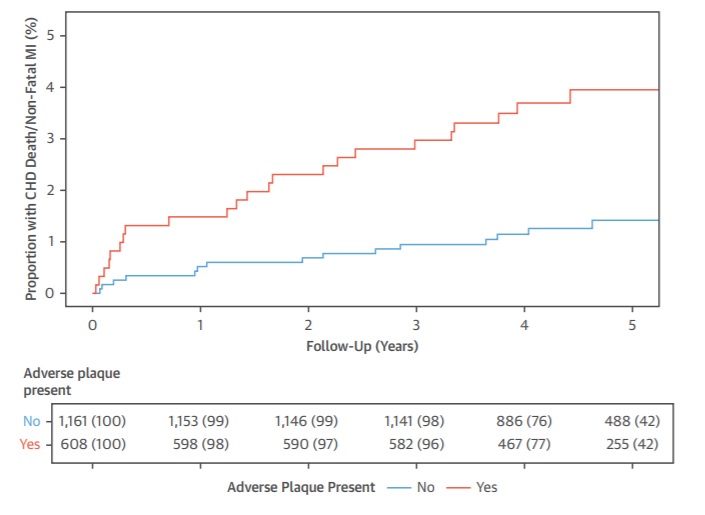


**References:**

1. Oikonomou EK, Antonopoulos AS, Schottlander D, et al. Standardized measurement of coronary inflammation using cardiovascular computed tomography: Integration in clinical care as a prognostic medical device. *Cardiovasc Res*. 2021;117(13):2677-2690. doi:10.1093/cvr/cvab286

2. Xie JX, Cury RC, Leipsic J, et al. The Coronary Artery Disease–Reporting and Data System (CAD-RADS): Prognostic and Clinical Implications Associated With Standardized Coronary Computed Tomography Angiography Reporting. *JACC: Cardiovascular Imaging*. 2018;11(1):78-89. doi:10.1016/j.jcmg.2017.08.026

3. Hoffmann U, Moselewski F, Nieman K, et al. Noninvasive Assessment of Plaque Morphology and Composition in Culprit and Stable Lesions in Acute Coronary Syndrome and Stable Lesions in Stable Angina by Multidetector Computed Tomography. *J Am Coll Cardiol*. 2006;47(8):1655-1662. doi:10.1016/j.jacc.2006.01.041

4. Williams MC, Moss AJ, Dweck M, et al. Coronary Artery Plaque Characteristics Associated With Adverse Outcomes in the SCOT-HEART Study. *Journal of the American College of Cardiology*. 2019;73(3):291-301. doi:10.1016/j.jacc.2018.10.066
